# The Leucine-Responsive Regulatory Protein Lrp Participates in Virulence Regulation Downstream of Small RNA ArcZ in Erwinia amylovora

**DOI:** 10.1128/mBio.00757-19

**Published:** 2019-05-28

**Authors:** Jeffrey K. Schachterle, George W. Sundin

**Affiliations:** a Genetics Graduate Program, Michigan State University, East Lansing, Michigan, USA; b Department of Plant, Soil, and Microbial Sciences, Michigan State University, East Lansing, Michigan, USA; University of California, Berkeley

**Keywords:** FlhDC, Hfq, fire blight

## Abstract

Erwinia amylovora causes the devastating fire blight disease of apple and pear trees. During systemic infection of host trees, pathogen cells must rapidly respond to changes in their environment as they move through different host tissues that present distinct challenges and sources of nutrition. Growing evidence indicates that small RNAs (sRNAs) play an important role in disease progression as posttranscriptional regulators. The sRNA ArcZ positively regulates the motility phenotype and transcription of flagellar genes in E. amylovora Ea1189 yet is a direct repressor of translation of the flagellar master regulator, FlhD. We utilized transposon mutagenesis to conduct a forward genetic screen and identified suppressor mutations that increase motility in the Ea1189*ΔarcZ* mutant background. This enabled us to determine that the mechanism of transcriptional activation of the *flhDC* mRNA by ArcZ is mediated by the leucine-responsive regulatory protein, Lrp. We show that Lrp contributes to expression of virulence and several virulence-associated traits, including production of the exopolysaccharide amylovoran, levansucrase activity, and biofilm formation. We further show that Lrp is regulated posttranscriptionally by ArcZ through destabilization of *lrp* mRNA. Thus, ArcZ regulation of FlhDC directly and indirectly through Lrp forms an incoherent feed-forward loop that regulates levansucrase activity and motility as outputs. This work identifies Lrp as a novel participant in virulence regulation in E. amylovora and places it in the context of a virulence-associated regulatory network.

## Abstract (precis)

Fire blight disease continues to plague the commercial production of apples and pears despite more than a century of research into disease epidemiology and disease control. The causative agent of fire blight, Erwinia amylovora coordinates turning on or off specific virulence-associated traits at the appropriate time during disease development. The development of novel control strategies requires an in-depth understanding of E. amylovora regulatory mechanisms, including regulatory control of virulence-associated traits. This study investigates how the small RNA ArcZ regulates motility at the transcriptional level and identifies the transcription factor Lrp as a novel participant in the regulation of several virulence-associated traits. We report that ArcZ and Lrp together affect key virulence-associated traits through integration of transcriptional and posttranscriptional mechanisms. Further understanding of the topology of virulence regulatory networks can uncover weak points that can subsequently be exploited to control E. amylovora.

## INTRODUCTION

Erwinia amylovora is the causative agent of fire blight, a devastating disease of apple and pear trees. E. amylovora cells are disseminated to flowers of new hosts by wind, rain, or insects (1), where under optimal conditions, they grow on the stigma to population levels of 10^6^ to 10^7^ cells per flower (2). To spread systemically through the host, the cells must reach the host vascular tissue by invading natural openings in the hypanthium (3). To reach the hypanthium, E. amylovora cells must swim through nectar, as the osmotic stress from the concentrated sugar in nectar prevents cell division (4). Flagellar motility is a virulence factor with the critical role of enabling E. amylovora cells to swim through nectar to the hypanthium (5, 6). Upon reaching the hypanthium, E. amylovora utilizes a type III secretion system to translocate effector proteins into host cells; these effectors function to suppress host defenses and cause host cell death through unknown mechanisms (7, 8). During systemic infection of hosts, E. amylovora cells can also enter the host vascular tissue where three exopolysaccharides, amylovoran (9), levan (10), and cellulose (11), contribute to biofilm development and significant population expansion that occludes the host xylem vessels (12). These populations spread systemically through the host and can also emerge from host tissues as ooze droplets that function in the dispersal of E. amylovora to new hosts (13).

Flagella are motility appendages that span the inner and outer membranes of Gram-negative bacteria, and the genes coding for flagellar structures tend to be organized in clusters of operons in the bacterial genome. In the Enterobacteriaceae, coordinated transcriptional control of flagellar structural genes is accomplished through the flagellar master regulators FlhD and FlhC, which are cotranscribed in an operon. Together, FlhD and FlhC form a complex that will activate expression of early- and middle-class flagellar genes, including *fliA*, the flagellar sigma factor. This regulatory cascade has been studied in detail and has been thoroughly reviewed (14, 15). Although FlhD and FlhC act as master regulators of flagellar biosynthesis, transcriptional control of the *flhDC* operon is complex (16). In Escherichia coli, several transcription factors are known to directly control transcription of the *flhDC* operon, including the catabolite repressor protein Crp (17), the histone-like nucleoid structuring protein H-NS (17), outer membrane protein regulator OmpR (18), lysR homolog A LrhA (19), and the regulator of capsule synthesis Rcs phosphorelay (20). In addition to these, other transcription factors are known to have indirect effects on the *flhDC* operon (21).

In E. amylovora, transcription of the *flhDC* operon is known to be regulated directly by the Rcs phosphorelay (22). Additional transcription factors are also known to affect the motility phenotype as well but have not yet been demonstrated to regulate *flhDC*. These additional factors contributing to motility include ArcA and OmpR (23), HrpL, the alternative sigma factor involved in transcription of type III secretion system genes (24), CsrA (25), and the EnvZ/OmpR two-component system (26). The effect of HrpL on motility suggests a link between flagellar motility and regulation of other virulence-associated traits. In support of this, flagellar genes were expressed at higher levels in susceptible hosts than in less-susceptible hosts (6). However, no role for *flhDC* in the expression of virulence-associated traits outside flagellar regulation has been reported. Other environmental conditions known to affect flagellar motility in E. amylovora include temperature and oxygen availability (27).

In addition to transcriptional control, *flhDC* mRNA is also subject to posttranscriptional control. The carbon storage regulator protein, CsrA, binds to *flhDC* mRNA to modulate translation posttranscriptionally (28), and several small regulatory RNAs (sRNAs) that are dependent on the chaperone protein Hfq control *flhDC* posttranscriptionally. In Escherichia coli, the sRNAs ArcZ, McaS, OmrA, OmrB, and OxyS all regulate *flhDC* posttranscriptionally (29, 30), and in E. amylovora, the sRNAs ArcZ and OmrAB regulate *flhDC* posttranscriptionally (31).

The *trans*-acting Hfq-dependent sRNA ArcZ negatively affects *flhDC* posttranscriptionally in both E. amylovora and Escherichia coli, and ArcZ acts as a negative regulator of flagellar motility in Escherichia coli (30, 32). In contrast, ArcZ is an activator of flagellar motility in E. amylovora (33, 34), and we recently reported that ArcZ positively affects the transcription of *flhDC* (31). We hypothesized that an additional factor affecting *flhDC* transcriptionally must be regulated by ArcZ in E. amylovora in addition to the known posttranscriptional interaction between ArcZ and *flhDC* mRNA. Owing to the large number of transcription factors already known to affect transcription of the *flhDC* operon, we utilized a forward genetic screen to identify candidate regulators that could connect ArcZ and transcription of the *flhDC* operon.

In this study, we conducted a suppressor screen by mutagenizing an E. amylovora Δ*arcZ* mutant with a transposon to generate insertional mutants and screened those mutants for swimming motility to identify mutants that restored motility in the low-motility *ΔarcZ* genetic background. The purpose of this mutant screen was to identify candidate regulatory factors that act between ArcZ and flagellar motility that can reconcile the contradiction between ArcZ activation and repression of *flhDC* at the transcriptional and posttranscriptional levels, respectively. We found that mutation of the leucine-responsive regulatory protein (Lrp) in the *ΔarcZ* mutant background reversed the loss of motility and conferred hypermotility. Lrp is a broadly conserved global transcription factor that responds to nutrient levels by directly binding to leucine and controls the expression of amino acid biosynthesis genes (35). The Lrp regulon and DNA binding are both altered by binding of leucine (36, 37). Lrp has also been associated with the expression of virulence-associated genes, especially those in control of fimbriae (38–41), and is an activator of motility (42, 43). Mutants lacking Lrp have reduced virulence in Vibrio vulnificus and Xenorhabdus nematophila (43, 44), but a Δ*lrp* mutant in Salmonella enterica serovar Typhimurium was fully virulent (45). In E. amylovora, we found that in addition to repressing flagellar motility, Lrp modulates several virulence-associated traits and overall virulence, thus acting as a novel participant in virulence regulation in E. amylovora.

## RESULTS

### Suppressor screen to identify candidate regulators acting between ArcZ and *flhDC*.

**FIG 1:**
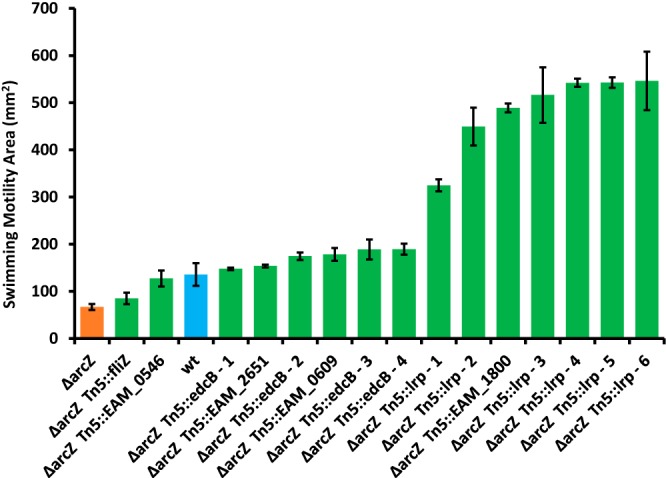
Swimming motility of suppressor Tn mutants. Shown are mutants resulting from Tn*5* mutagenesis of the E. amylovora Ea1189Δ*arcZ* mutant that were selected as motility suppressors and for which the Tn insertion site was successfully identified by sequencing. Blue bar, wild-type strain (wt); orange bar, Δ*arcZ* mutant. Green bars represent Tn mutants with a significant (*P* < 0.05) increase in motility compared to that of the Δ*arcZ* mutant by Student’s *t* test. Error bars represent standard deviations, and the experiment was repeated 4 times.

To identify novel targets of ArcZ that participate in the regulation of flagellar motility, we conducted transposon (Tn) mutagenesis in the E. amylovora Ea1189*ΔarcZ* mutant strain and screened for suppressor mutants that restored swimming motility. In a screen of 18,000 Tn mutants, we selected 27 mutants with consistently greater swimming motility than the parental *ΔarcZ* mutant strain. Of these, flanking regions of 15 were sequenced using arbitrary PCR. For the remaining mutants, Sanger sequencing either failed in three independent attempts or gave disagreeing results from one side of the Tn compared to the other. These cases may represent multiple insertions or genomic rearrangements as a result of the Tn mutagenesis. The locations of the Tn insert from successfully sequenced mutants with single insertions appear in Table 1. The most common gene to be interrupted by Tn insertion was the leucine-responsive regulatory protein (Lrp), with six independent mutants recovered with insertions in the 5′ untranslated region or coding region. Mutants were also obtained with insertions in the diguanylate cyclase *edcB*, which functions in the synthesis of the second messenger molecule bis-(3′,5′)-cyclic diguanosine monophosphate (c-di-GMP) that is linked to motility in E. amylovora (46), as well as in *fliZ*, a regulator of the flagellar sigma factor, FliA (47). We additionally recovered mutants with Tn insertions in a number of genes encoding proteins with poorly characterized functions, including one (EAM_2652) located in a type III secretion gene cluster that is not involved in virulence in an immature pear infection model (48). Each of these mutants exhibited increased motility relative to the parental Δ*arcZ* mutant strain, with some Tn insertions restoring wild-type levels of swimming motility and others conferring hypermotility (Fig. 1). One of the mutants with a Tn insertion in the protein-coding region of *lrp* had reduced motility compared to that of the other mutants with *lrp* insertions, with no obvious explanation for this difference based on the site of the Tn insertion; nonetheless, this insertional mutant of *lrp* was still hypermotile compared to the wild-type strain. The Tn insertions that conferred hypermotility were those with insertions in *lrp* or in EAM_1800, which codes for a putative phage protein.

**TABLE 1:** Locations and predicted functions of transposon insertions from the suppressor screen

Locus tag	Gene name (corr. no.)^*a*^	Tn insertion site^*b*^	Annotated function^*c*^
EAM_0546		616041	Hypothetical protein
EAM_0564	*edcB* (3)	629901	Diguanylate cyclase
EAM_0564	*edcB* (4)	629858	Diguanylate cyclase
EAM_0564	*edcB* (1)	630715	Diguanylate cyclase
EAM_0564	*edcB* (2)	630942	Promoter region of diguanylate cyclase *edcB*
EAM_0609		685056	Putative acyltransferase
EAM_1328	*lrp* (5)	1439553	5′ UTR of leucine-responsive regulatory protein, global transcription factor
EAM_1328	*lrp* (2)	1439574	5′ UTR of leucine-responsive regulatory protein, global transcription factor
EAM_1328	*lrp* (3)	1439605	5′ UTR of leucine-responsive regulatory protein, global transcription factor
EAM_1328	*lrp* (6)	1439650	Leucine-responsive regulatory protein, global transcription factor
EAM_1328	*lrp* (1)	1439694	Leucine-responsive regulatory protein, global transcription factor
EAM_1328	*lrp* (4)	1439784	Leucine-responsive regulatory protein, global transcription factor
EAM_1800		1939771	Phage protein
EAM_2064	*fliZ*	2218479	Putative regulator of sigma-F (sigma 28)
EAM_2652		2890719	Hypothetical protein in type 3 secretion system cluster II

a Corr no., correlation number refers to identifying number shown in Fig. 1 to correlate specific insertions to corresponding motility measurements.

b Nucleotide position based on the ATCC 49946 genome (73; GenBank accession no. GCA_000027215.1).

c Based on annotation of the ATCC 49946 genome (73; GenBank accession no. GCA_000027215.1).

### Lrp is a motility regulator.

**FIG 2:**
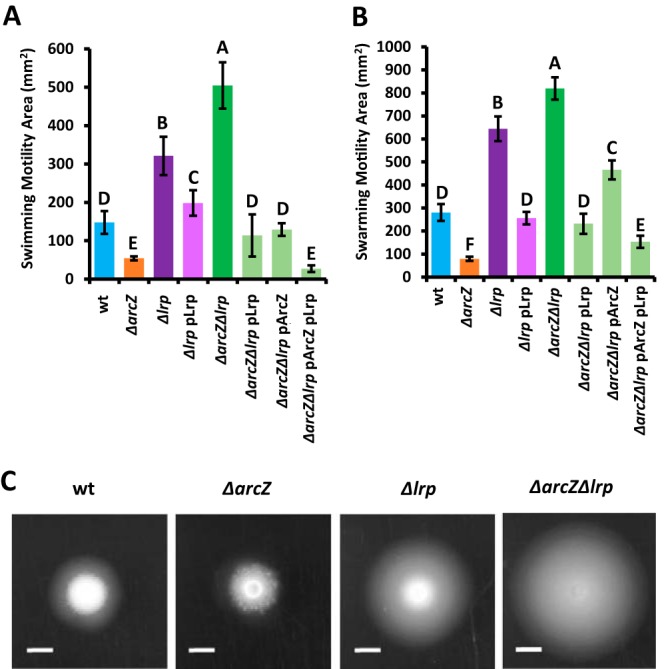
Lrp is a motility regulator epistatic to ArcZ. Swimming (A) and swarming (B) motility of indicated strains grown in or on the surface of soft agar medium, respectively. Error bars represent standard deviations, and groups with shared uppercase letter designations do not differ significantly (*P* > 0.05) from each other by Tukey’s honestly significant difference (HSD) test. (C) Representative images of swarming colonies after 48 h of incubation at 28°C. Scale bars, 3 mm.

We generated site-directed mutants of *lrp* in both Ea1189 wild-type and *ΔarcZ* mutant backgrounds. Assessment of swimming motility confirmed that Lrp is a motility regulator in E. amylovora. We found similar trends when strains were assessed for movement across a surface, or swarming motility. Swimming motility after 24 h of incubation is shown in Fig. 2A. Swarming motility after 48 h of incubation is shown in Fig. 2B, with representative swarming colonies shown in Fig. 2C. As previously known (34), loss of *arcZ* reduced motility relative to that of the wild type. Deletion of *lrp* conferred hypermotility, and deletion of both *arcZ* and *lrp* resulted in even greater motility than deletion of *lrp* alone. Wild-type levels of swarming motility were restored in the Δ*lrp* mutant when *lrp* was provided on a plasmid with its native promoter, which only partially complemented for swimming motility. When *lrp* was provided on a plasmid to the Δ*arcZ* Δ*lrp* double mutant, the motility was similar to that of wild-type cells. Provision of *arcZ* on a plasmid in the Δ*arcZ* Δ*lrp* double mutant resulted in a reduction of motility relative to that in the Δ*arcZ* Δ*lrp* double mutant and similar to wild-type swimming motility, but swarming motility was still greater than that that in the wild type. When both *arcZ* and *lrp* were provided on plasmids to the Δ*arcZ* Δ*lrp* double mutant, the resulting motility was lower than the wild type and similar to the Δ*arcZ* mutant. In these tests of complementation, similar trends were observed overall in tests of both swimming and swarming motility.

### Lrp regulates *flhD*C and flagellin transcript abundance.

**FIG 3:**
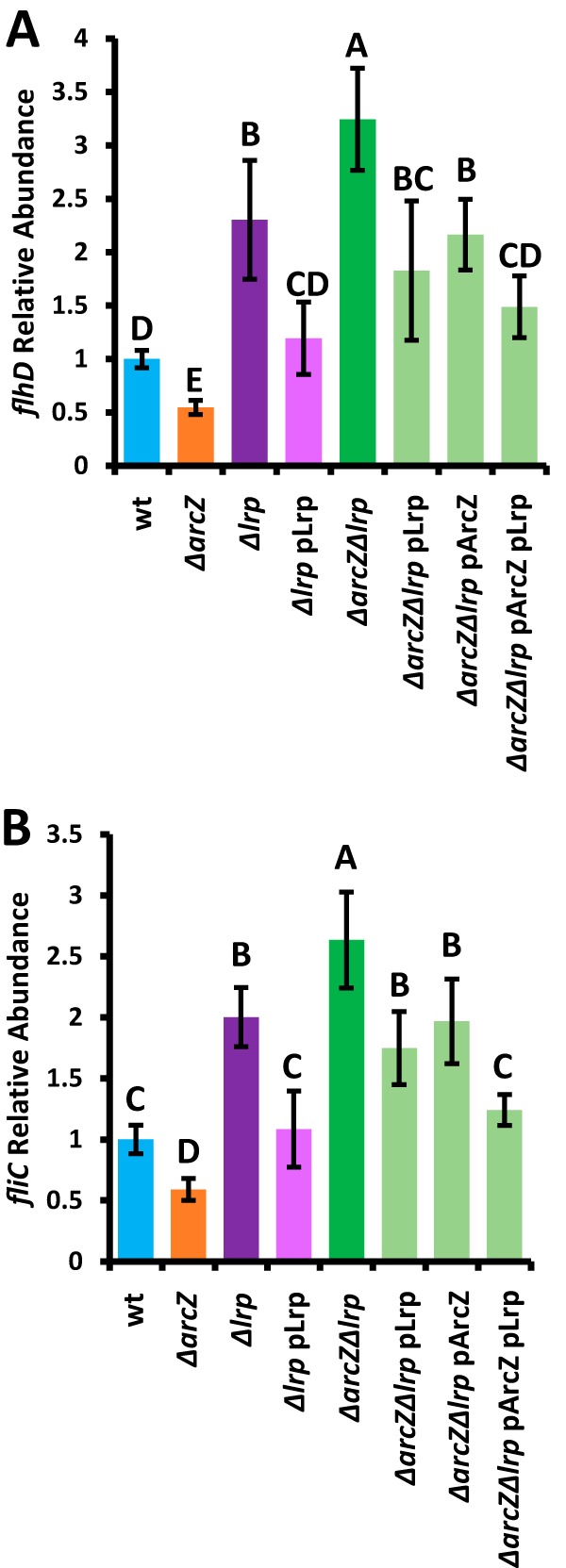
Lrp regulates abundance of flagellar transcripts epistatic to ArcZ. Relative abundance of *flhDC* (A) or *fliC* (B) transcripts as determined by quantitative real-time PCR using *recA* as an endogenous control. The experiment was repeated three times, and error bars represent standard deviations. Groups with shared uppercase letter designations do not differ significantly (*P* > 0.05) from each other by Tukey’s HSD test.

Because deletion of *arcZ* reduced *flhDC* mRNA abundance in E. amylovora Ea1189 (31), we hypothesized that loss of *lrp* acts as a suppressor mutation of Δ*arcZ* by restoring *flhDC* transcript levels. To test this hypothesis, we conducted quantitative real-time PCR analyses to quantify transcript levels in the Δ*lrp* mutants. We found that loss of *lrp* increased *flhDC* transcript abundance relative to that in the wild type and that this increase also occurred in the *ΔarcZ* genetic background, indicating that Lrp is epistatic to ArcZ for the regulation of flagellar motility (Fig. 3A). The increased *flhDC* mRNA abundance was fully complemented in the *Δlrp* mutant and partially complemented in the *ΔarcZ Δlrp* double mutant by providing *lrp* on a plasmid with its native promoter. Provision of *arcZ* on a plasmid complemented *flhDC* mRNA levels in the *ΔarcZ Δlrp* double mutant to *flhDC* mRNA levels in the *Δlrp* mutant. We additionally tested mRNA abundance of *fliC*, which codes for flagellin, and found similar trends (Fig. 3B).

### ArcZ regulates *lrp* posttranscriptionally and does not affect *lrp* transcript levels.

**FIG 4:**
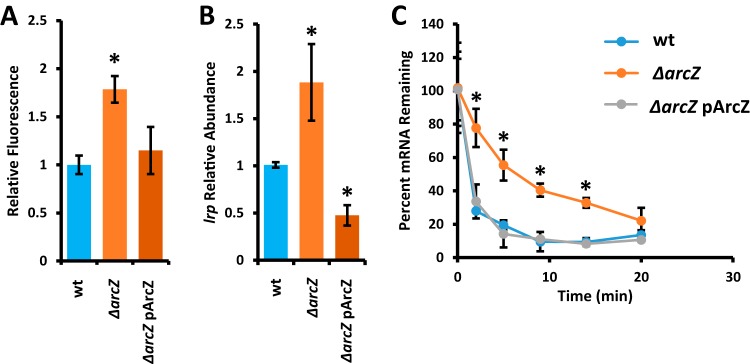
ArcZ regulates Lrp posttranscriptionally by destabilizing *lrp* mRNA. (A) Relative fluorescence of strains carrying the Lrp translational fusion construct. (B) Relative abundance of *lrp* mRNA as determined by quantitative real-time PCR. (C) Lrp transcript stability following addition of rifampin at time zero. All experiments were conducted at least three times, and error bars represent standard deviations. *, *P* < 0.05 compared to wild-type by Student’s *t* test.

Because Lrp is epistatic to ArcZ for *flhDC* expression, it must play its regulatory role between ArcZ and *flhDC* in the regulatory cascade. We tested the hypothesis that ArcZ regulates Lrp posttranscriptionally by constructing a translational fusion of the 5′ untranslated region (UTR) of *lrp* fused to green fluorescent protein (GFP). The 5′ UTR region was identified by conducting a rapid amplification of cDNA ends (5′ RACE) experiment to determine the transcriptional start site of *lrp*, which was located 103 nucleotides upstream from the start codon. When the translational fusion was compared between wild-type and *ΔarcZ* mutant cells, the fluorescence was higher in the *ΔarcZ* mutant than in the wild type, indicating posttranscriptional repression by ArcZ (Fig. 4A). This effect was complemented by providing *arcZ* on a plasmid with its native promoter.

Because the posttranscriptional effect of ArcZ on the *lrp* translational fusion was modest, we tested *lrp* transcript levels in the *ΔarcZ* mutant relative to that in the wild type to determine if ArcZ also regulates *lrp* at the transcriptional level. Quantitative real-time PCR revealed an increase in *lrp* transcript abundance in the *ΔarcZ* mutant relative to that in the wild type, which was complemented by provision of ArcZ by a plasmid (Fig. 4B). To test whether this regulatory effect was at the level of transcriptional control, we generated a transcriptional fusion reporter with the *lrp* promoter upstream of *gfp*. Comparing between the wild type and the *ΔarcZ* mutant, we found no difference in *lrp* promoter activity (data not shown).

Because transcript levels were increased but promoter activity was unaffected, we tested *lrp* transcript stability in wild-type and Δ*arcZ* mutant cells following inhibition of transcription in cells by the addition of rifampin. We found that in wild-type cells, *lrp* transcripts had a half-life of approximately 1.7 min and that *lrp* transcripts exhibited much greater stability in Δ*arcZ* mutant cells, with a half-life of approximately 6.1 min (Fig. 4C). Together, these data indicate that ArcZ acts a negative regulator of Lrp by posttranscriptionally destabilizing *lrp* mRNA.

### Lrp affects additional virulence-associated traits.

Because Lrp is a global regulator of amino acid biosynthesis (49) and recent reports suggest it can affect one-third of genes in Escherichia coli (37), we tested the impact of *lrp* deletion on a variety of virulence-associated traits in both the E. amylovora Ea1189 wild-type and *ΔarcZ* genetic backgrounds.

**FIG 5:**
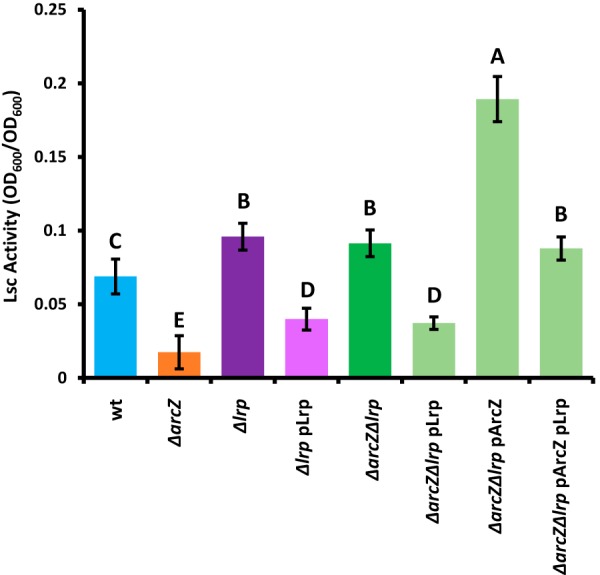
Lrp affects levansucrase activity epistatic to ArcZ. Levansucrase activity was assayed from overnight cultures by mixing culture supernatants in a 1:1 ratio with levansucrase assay buffer (phosphate-buffered 2 M sucrose) and incubating at 37°C for 24 h. Groups with shared uppercase letter designations do not differ significantly (*P* > 0.05) by Tukey’s HSD test. The experiment was repeated four times, and error bars represent standard deviations.

We first tested the impact of *lrp* on activity of levansucrase (Lsc), a secreted enzyme that uses sucrose as a substrate to form the exopolysaccharide levan, a homopolymer of fructose (50). The Lsc activities as found in various strains are shown in Fig. 5A. The *Δlrp* mutant had a slight increase in Lsc activity. The *ΔarcZ* mutant has reduced Lsc activity, but the *ΔarcZ Δlrp* double mutant had the same level of Lsc activity as the *Δlrp* single mutant. Providing *lrp* on a plasmid in either the *Δlrp* mutant or the *ΔarcZ Δlrp* double mutant reduced Lsc activity. Providing *arcZ* on a plasmid in the *ΔarcZ Δlrp* double mutant resulted in increased Lsc activity, but providing both *arcZ* and *lrp* on plasmids in the *ΔarcZ Δlrp* double mutant resulted in no difference. Because the Lsc enzyme uses sucrose as a substrate, Lsc activity correlates with colony morphology of cells grown on solid medium containing sucrose. Wild-type cells grown on solid medium containing sucrose formed domed colonies that grew over time, but the *ΔarcZ* mutant colonies were low and had a rougher texture, until after 3 to 5 days of growth, when a dome began to form in the center of the colony (see Fig. S1 in the supplemental material). The *Δlrp* mutant colonies started to dome, but rapidly lost form and spread across the surface of the medium. The *ΔarcZ Δlrp* double mutants reverted to the wild-type domed colony morphology. Provision of *lrp* on a plasmid in the *Δlrp* mutant or the *ΔarcZ Δlrp* double mutant resulted in a colony morphology similar to that of the *ΔarcZ* mutant (Fig. S1). Provision of *arcZ* or *arcZ* and *lrp* on plasmids in the *ΔarcZ Δlrp* double mutant resulted in spreading and runny colony morphology, similar to that of the *Δlrp* mutant.

10.1128/mBio.00757-19.1 FIG S1 ArcZ and Lrp affect colony morphology when cells grown on LB agar plates amended to 5% (wt/vol) sucrose. Strains grown at 28°C for indicated times and imaged using dissecting scope. Download FIG S1, PDF file, 0.3 MB. Copyright © 2019 Schachterle and Sundin. 2019 Schachterle and Sundin This content is distributed under the terms of the Creative Commons Attribution 4.0 International license.

**FIG 6:**
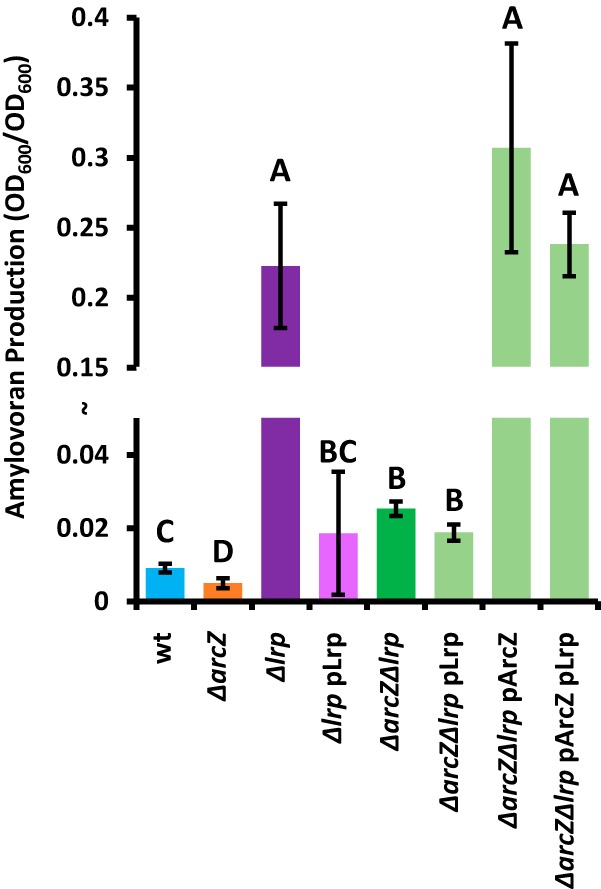
Lrp affects production of amylovoran epistatic to ArcZ. Amylovoran was quantified from supernatants of cultures grown in MBMA medium for 24 h by addition of cetylpyridinium chloride. Groups with shared uppercase letter designations do not differ significantly (*P* > 0.05) by Tukey’s HSD test. The experiment was repeated four times, and error bars represent standard deviations.

When assayed for the production of the exopolysaccharide amylovoran, we confirmed the previous finding that the *ΔarcZ* mutant is deficient for amylovoran production (34), but the *Δlrp* mutant had high levels of amylovoran production (Fig. 6). The *ΔarcZ Δlrp* double mutant had increased amylovoran production relative to that of the wild type but much less than the *Δlrp* single-deletion mutant. The *Δlrp* mutant was fully complemented by *lrp* provided on a plasmid, but the *ΔarcZ Δlrp* double mutant was only partially complemented by providing *lrp* on a plasmid. In the *ΔarcZ Δlrp* double mutant, providing either *arcZ* alone on a plasmid or *arcZ* and *lrp* both on plasmids resulted in high levels of amylovoran production, similar to those of the Δ*lrp* mutant.

**FIG 7:**
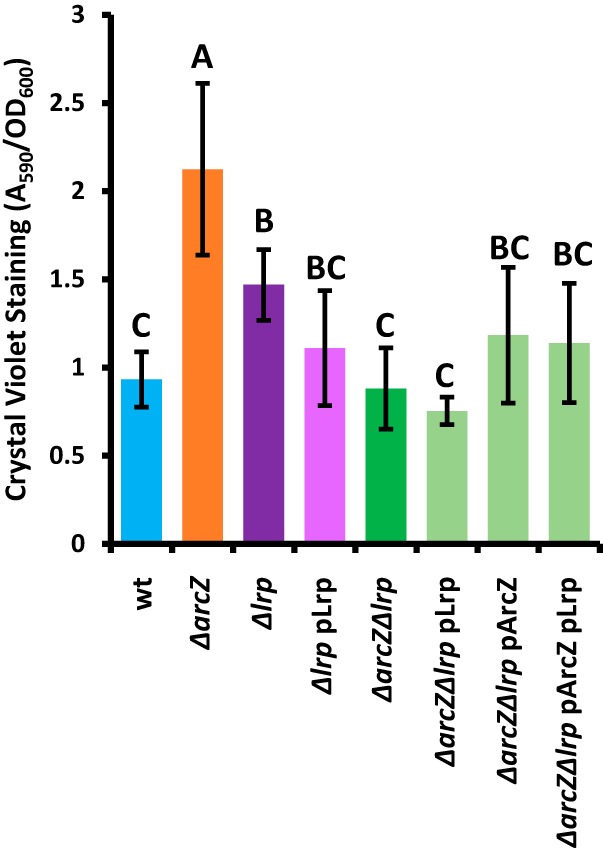
Lrp reverses high crystal violet staining of the E. amylovora Ea1189Δ*arcZ* mutant. Cells were grown in 96-well plates. After removal of planktonic cells, adherent cells were stained with crystal violet. Following rinsing of unbound crystal violet and drying, stain bound to adherent cells was solubilized by an ethanol-acetone solution, and the 590-nm absorbance was measured and normalized to the OD_600_ of the cultures. *, *P* < 0.05 compared to wild-type by Student’s *t* test.

Because we found that ArcZ and Lrp regulate the production of the exopolysaccharides amylovoran and levan and ArcZ regulates cell surface attachment (34), all of which contribute to crystal violet staining in a biofilm assay (12, 51), we hypothesized that *lrp* might also behave as an overall biofilm regulator. Consistent with previous findings (34), we found that the *ΔarcZ* mutant had higher than wild-type levels of crystal violet staining, despite its low levels of exopolysaccharide production (Fig. 7). The *Δlrp* mutant also had elevated levels of crystal violet staining relative to that of the wild type, but the *ΔarcZ Δlrp* double mutant did not differ from the wild type. Providing *lrp* on a plasmid in the *Δlrp* mutant background restored wild-type levels of crystal violet staining, but provision of *arcZ*, *lrp*, or both in the *ΔarcZ Δlrp* double mutant had no effect.

Because the loss of *arcZ* leads to reduced translocation of type III effector proteins as observable as a reduced hypersensitive response (HR) on nonhost tobacco (34), we tested whether *lrp* had any effect on HR in the wild-type or *ΔarcZ* mutant backgrounds. We found that loss of *lrp* had no effect on HR in tobacco (data not shown). The *Δlrp* single mutant had the same HR response as the wild type; the *ΔarcZ* mutant and the *ΔarcZ Δlrp* double mutant both exhibited the same reduced HR.

In summary, we found that Lrp regulates the production of the exopolysaccharides amylovoran and levan and crystal violet staining in a biofilm assay. In each of these virulence-associated phenotypes, loss of *lrp* in the *ΔarcZ* mutant background reversed the effect of the loss of *arcZ*, suggesting that Lrp is acting epistatically to ArcZ in the regulation of these traits. However, Lrp appears to have no effect on type III secretion, as loss of *lrp* in wild-type or *ΔarcZ* mutant strains did not affect the hypersensitive response in nonhost tobacco.

### Lrp participates in general virulence regulation.

**FIG 8:**
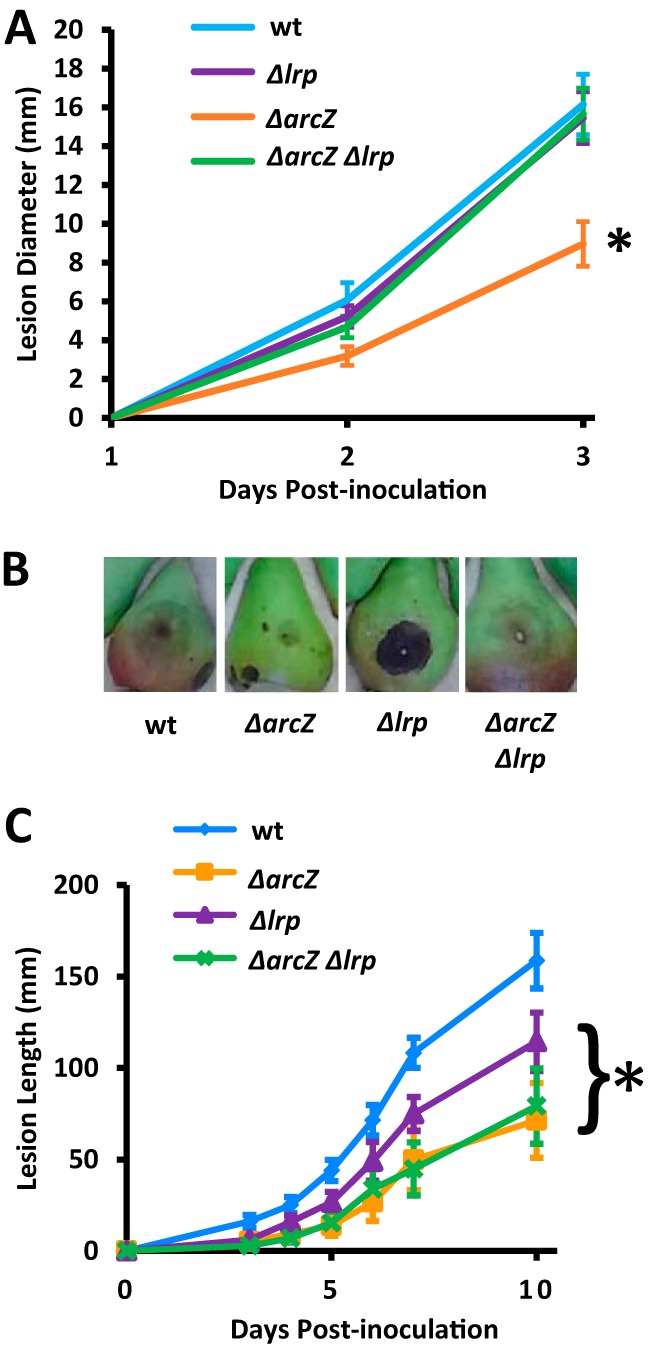
Lrp participates in overall virulence regulation. (A) Immature pears were inoculated with 10^3^ cells of E. amylovora strains and incubated at 28°C under high humidity conditions. Diameters of disease lesions on pears were measured every 24 h. (B) Representative pictures of inoculated immature pears. (C) Apple shoots on potted trees were cut inoculated with scissors dipped in a bacterial suspension of 5 × 10^8^ CFU/ml. Lesion length from the point of inoculation was measured at indicated time points. *, *P* < 0.05 compared to wild-type by Student’s *t* test. Experiments were repeated at least twice, with at least six replicates per experiment.

Because the loss of *lrp* acts as a suppressor mutation for several ArcZ-regulated and virulence-associated phenotypes, we examined the overall effect of Lrp on virulence in both the E. amylovora Ea1189 wild-type and *ΔarcZ* mutant backgrounds. We conducted tests using both an immature pear fruit model of infection (52) and an apple shoot infection model (12). We found that loss of *lrp* in the wild-type background had no effect on virulence in immature pears and that Δ*arcZ* mutants had reduced virulence relative to that of the wild type at 2 and 3 days postinoculation (Fig. 8A). However, loss of *lrp* in the Δ*arcZ* genetic background restored wild-type levels of virulence. Images of representative infected pears are shown in Fig. 8B, in which reduced lesion size is only observed in the *ΔarcZ* mutant; however, the lesions in immature pears inoculated with the Δ*lrp* mutant showed much greater incidence of necrosis rather than the water-soaking symptom that was predominant in the other strains.

In the apple shoot infection model, we found that all of the mutants tested had reduced lesion length from the point of inoculation relative to that of the wild type from 4 days postinoculation through 10 days postinoculation (Fig. 8C). The Δ*arcZ* and Δ*arcZ* Δ*lrp* mutants had the same rates of disease progression, and the Δ*lrp* single-deletion mutant had intermediate virulence that was reduced relative to that of the wild type.

### *flhDC* expression in Δ*arcZ* restores phenotypes.

**FIG 9:**
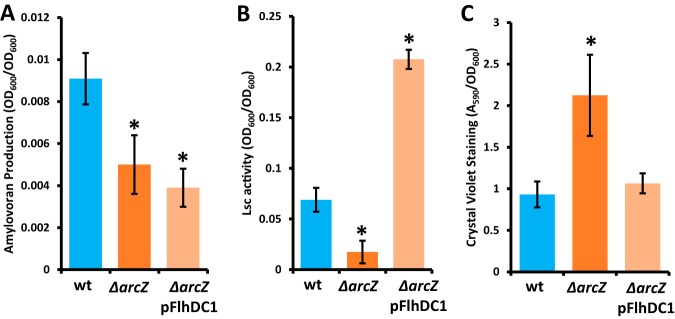
Complementation of the E. amylovora Ea1189Δ*arcZ* mutant with *flhDC* and its effects on amylovoran (A), levansucrase (B), and crystal violet staining (C) phenotypes. Experiments were repeated 4 times. *, *P* < 0.05 relative to wild-type phenotypes by Student’s *t* test.

Because the flagellar motility, amylovoran, levan, biofilm, and virulence phenotypes of the Δ*arcZ* mutant were all reversed by also deleting *lrp*, we hypothesized that increased expression of *flhDC* may be sufficient to restore these phenotypes in the Δ*arcZ* mutant background. To test this hypothesis, we assayed motility, exopolysaccharide production, and biofilm formation in the Δ*arcZ* deletion mutant expressing additional *flhDC* from a plasmid. We found that *flhDC* on a plasmid in the Δ*arcZ* mutant background conferred motility greater than that of the wild type in both swimming and swarming motility assays (see Fig. S2). When tested for exopolysaccharide formation, we found that provision of *flhDC* on a plasmid had no effect on amylovoran biosynthesis (Fig. 9A) but did increase levansucrase activity (Fig. 9B). Additionally, provision of *flhDC* on a plasmid in the Δ*arcZ* mutant background led to an intermediate colony morphology when cells were grown on medium containing sucrose (Fig. S1). Additionally, providing *flhDC* on a plasmid in the Δ*arcZ* mutant background in a biofilm assay restored wild-type levels of crystal violet staining (Fig. 9C). These data demonstrate that restoring *flhDC* is sufficient to restore several virulence-associated traits in the E. amylovora Δ*arcZ* mutant.

10.1128/mBio.00757-19.2 FIG S2 Providing *flhDC1* on a plasmid with native promoter is sufficient to restore swimming (A) and swarming (B) motility to the *arcZ* mutant. For swimming, cells were stab inoculated on soft agar (0.25% agar) and incubated at 28°C for 24 h. For swarming, cells were dripped onto the surface of soft agar (0.3% agar) and incubated at 28°C for 48 h. Motility halos were imaged and area covered quantified using ImageJ. *, *P* < 0.05 compared to wild-type by Student’s *t* test. Motility tests were repeated at least 4 times. Download FIG S2, PDF file, 0.2 MB. Copyright © 2019 Schachterle and Sundin. 2019 Schachterle and Sundin This content is distributed under the terms of the Creative Commons Attribution 4.0 International license.

## DISCUSSION

In this study, we used a suppressor screen to understand how the Hfq-dependent sRNA ArcZ is regulating flagellar motility at the transcriptional level. This led us to determine that ArcZ posttranscriptionally regulates *lrp* mRNA stability and that *lrp* acts epistatically to ArcZ in the regulation of *flhDC* transcript abundance. We further determined that *lrp* participates in general virulence regulation and that *flhDC* of E. amylovora affects the virulence-associated traits of levansucrase activity, amylovoran production, and biofilm formation in addition to flagellar motility.

We have identified Lrp as a negative regulator of *flhDC* transcript abundance, and this regulation could be through a direct interaction between Lrp and the *flhDC* promoter or indirectly through another transcriptional or posttranscriptional regulator. The fact that we did not identify any other known regulators of *flhDC* in our transposon mutant screen suggests that it may be a direct interaction, but the screen was not necessarily saturating. Among known targets of Lrp that could explain the observed regulatory effects on *flhDC* as well as on other virulence-associated traits is LrhA, because chromatin immunoprecipitation sequencing (ChIP-seq) experiments conducted in Escherichia coli indicated that Lrp bound to the promoter of *lrhA* but not *flhDC* (37). LrhA is a known direct regulator of *flhDC*, and its homologs are known regulators of a variety of virulence and virulence-associated traits in several species of Enterobacteriaceae (53–57). Homologs of LrhA include HexA in Pectobacterium carotovorum and PecT in Dickeya dadantii, two plant pathogens closely related to Erwinia amylovora. LrhA or LrhA homologs affect virulence or virulence-associated traits in the genera *Dickeya* (54), *Escherichia* (19, 57), *Pantoea* (56), *Pectobacterium* (53), and *Xenorhabdus* (55). To better understand this regulatory cascade, additional work is needed to determine if Lrp regulation of *flhDC* is direct or indirect through another regulator such as LrhA. While Lrp, along with LrhA and its homologs, regulates virulence-associated traits in the Enterobacteriaceae, *lrp* mutants of *Salmonella* Typhimurium are not impaired in virulence (45), but *lrp* mutants of E. amylovora and Xenorhabdus nematophila are virulence impaired (43). This suggests that although Lrp is broadly conserved, it appears to have evolved divergent roles in virulence across the Enterobacteriaceae, consistent with the divergence in the hosts of these pathogens.

We found that in E. amylovora, Lrp affects the biosynthesis of the exopolysaccharides amylovoran and levan as well as biofilm formation by using a crystal violet staining assay. Our findings suggest that Lrp has weak negative effects on levansucrase activity and a strong negative effect on amylovoran biosynthesis. However, expression of *arcZ* in the Δ*arcZ Δlrp* double mutant increased both levansucrase activity and amylovoran biosynthesis, suggesting that although Lrp is affecting exopolysaccharide production downstream of ArcZ, ArcZ has regulatory effects on exopolysaccharide production that are independent of Lrp. Although we found *arcZ* and *lrp* affect the exopolysaccharide formation phenotypes, further work is required to determine whether these effects may be due to direct or indirect regulation at the transcriptional, posttranscriptional, or posttranslational level. In spite of low exopolysaccharide production, the Δ*arcZ* mutant has high crystal violet staining in this assay due to surface hyperattachment (34). The Δ*lrp* mutant also has elevated levels of crystal violet staining, but loss of both *arcZ* and *lrp* returned crystal violet staining to wild-type levels. In Escherichia coli, Lrp is a direct regulator of type I fimbria expression by binding to the promoter region of *fimA* (39); however, in Erwinia amylovora, the type I fimbrial genes and promoters are poorly conserved (51). We hypothesize that the observed effects on biofilm by Lrp in the Δ*lrp* and Δ*arcZ Δlrp* mutants are due to changes in exopolysaccharide formation and motility and that any effect on fimbrial attachment structures is likely due to an indirect effect.

Although we found effects on exopolysaccharide production and biofilm formation, we found no effect from Lrp on the hypersensitive response, suggesting that Lrp does not affect secretion or translocation of effector proteins by the type III secretion system. The Δ*lrp* single mutant had an HR similar to that of wild-type cells, and there was no difference between the Δ*arcZ* mutant and the Δ*arcZ Δlrp* double mutant, as neither of these strains elicited HR at the cell densities tested. Although there was no observed effect on the type III secretion system, loss of *lrp* in the Δ*arcZ* mutant background was sufficient to restore full virulence in the immature pear infection model, where the Δ*arcZ* mutant has reduced virulence compared to that of the wild type. When inoculated into apple shoots, however, the loss of *lrp* was unable to restore any virulence in the Δ*arcZ* mutant background, and the Δ*lrp* single mutant had reduced virulence compared to that of the wild type. This indicates that proper regulation of virulence traits by Lrp is essential for full virulence in apple shoots but is dispensable for virulence in immature pears. Furthermore, it suggests that although the type III secretion system has reduced effector translocation in the Δ*arcZ* mutant, it is still enough for successful infection of immature pears when other virulence factors, such as amylovoran and levan, are being expressed at high enough levels. Altogether, this suggests that Lrp contributes to regulation of virulence and virulence-associated traits in E. amylovora, and that further elucidation of the mechanisms of this role in regulation will enhance the understanding of how E. amylovora integrates environmental cues to properly express these virulence-associated traits during disease development.

We further found thatFlhDC affects virulence-associated traits other than flagellar motility. Providing *flhDC* on a plasmid in the Δ*arcZ* mutant strain was sufficient to restore wild-type levels of levansucrase activity and crystal violet staining in a biofilm assay. Expression of *flhDC* in the Δ*arcZ* mutant background, however, did not restore amylovoran production. This coupled with the strong effect of *lrp* deletion on amylovoran production suggests that the ArcZ and Lrp effects on amylovoran are independent of FlhDC. These findings suggest that FlhDC may have additional specific roles in virulence trait regulation rather than only as a dedicated flagellar regulator. Future studies to characterize the FlhDC regulon in E. amylovora will aid in the determination of what other virulence-associated effects FlhDC may have in E. amylovora. FlhDC is known to play a role in regulating type III secretion in Dickeya dadantii (58), and a recent transcriptomic study in E. amylovora found that flagellar genes are expressed at higher levels in more-susceptible hosts than in less-susceptible hosts (6), which could also indicate a link between flagellar regulation and virulence signaling. These observations suggest that the FlhDC regulatory complex could include targets outside flagellar genes that explain these effects, as has been found in Dickeya dadantii (58). Another possible explanation is that the flagellum itself acts as a mechanosensor leading to another signal transduction pathway regulating virulence-associated phenotypes. Although not fully understood mechanistically, flagellar mechanosensing has been shown to result in altered motor-statin output (59) and could have farther reaching indirect effects on several cell processes.

Other studies conducted in Escherichia coli have found that Lrp acts as a hub for Hfq-dependent sRNA regulation. For example, the Hfq-dependent sRNA MicF interacts directly with *lrp* mRNA to repress translation (60). Other Hfq-dependent sRNAs, GcvB and DsrA, have also been shown to be posttranscriptional repressors of *lrp* in Escherichia coli (61, 62). However, ArcZ was found to have no posttranscriptional effects on Lrp in Escherichia coli (62). E. amylovora has a GcvB homolog but no homologs of MicF or DsrA. Here, we demonstrated that ArcZ also acts as a posttranscriptional regulator of *lrp* in E. amylovora by destabilizing *lrp* mRNA. This regulation is likely unique to E. amylovora, as ArcZ acts as a negative regulator of flagellar motility in other Enterobacteriaceae (32) but is an activator of flagellar motility by transcriptionally activating *flhDC* in E. amylovora (31). Although we have demonstrated that ArcZ posttranscriptionally regulates *lrp* in E. amylovora, additional work is needed to confirm that this interaction is direct and to identify the base-pairing interactions that lead to this effect.

**FIG 10:**
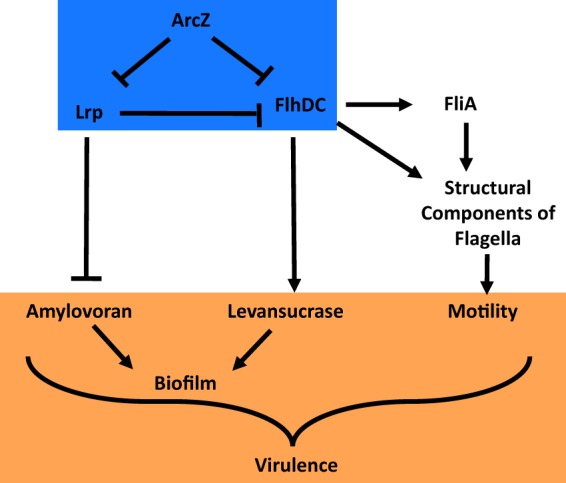
Proposed model of ArcZ and Lrp regulation of virulence-associated traits in E. amylovora. ArcZ regulates *flhDC* directly and indirectly through Lrp in an incoherent feed-forward loop (blue box). As the target of the loop, FlhDC regulates motility and levansucrase activity as outputs. ArcZ affects amylovoran production through Lrp, but amylovoran production is not affected by FlhDC. Lrp modulation of amylovoran and indirect effects on motility and levansucrase activity through FlhDC result in Lrp playing a role in general virulence regulation (orange box).

The regulation of *flhDC* both directly and indirectly by ArcZ forms an incoherent feed-forward loop (Fig. 10, blue box). Other sRNAs are known to participate in regulatory feed-forward loops. For example, in Escherichia coli, the iron-responsive transcription factor Fur represses transcription of superoxide dismutase (SodA) through a direct interaction (63, 64). However, Fur also represses transcription of the sRNA RyhB, which in turn acts as a negative regulator of SodA posttranscriptionally (65). In this way, Fur directly represses SodA while indirectly activating it, where the sRNA RyhB is the intermediate for the indirect interaction. ArcZ regulation of *flhDC* in E. amylovora is similar in that ArcZ acts as a direct repressor of *flhDC* and as an activator through an indirect pathway, forming an incoherent feed-forward loop. However, in the case of ArcZ, the sRNA is sitting at the head of the feed-forward loop rather than as the intermediate of the indirect regulation, as is the case for RyhB. In the ArcZ-*flhDC* feed-forward loop, we have identified Lrp as an intermediate for the indirect regulation. As a result, in the ArcZ-*lrp*-*flhDC* feed-forward loop, the direct interaction is via posttranscriptional repression, and the indirect activation is through transcriptional activation by ArcZ alleviating *flhDC* repression by repressing *lrp*. Coherent feed-forward loops in which a regulator activates directly and activates indirectly (or represses directly and represses indirectly) are approximately twice as abundant in Escherichia coli as in incoherent feed-forward loops (66). The utility of incoherent feed-forward loops is in the generation of a unique-shaped output pulse in the target gene in response to variations in the regulator at the head of the loop (67). The specific shapes of these are determined by interaction kinetics and the strength of the affinity between each of the players in the feed-forward loop. In general, incoherent feed-forward loops accelerate the response in the output, whereas coherent feed-forward loops have a low rate of response from signal to output (66). Further work is needed to determine the kinetics of how the ArcZ-*lrp*-*flhDC* feed-forward loop transforms changes in ArcZ abundance into changes in FlhDC abundance in E. amylovora. Because flagellar motility is of greatest importance to E. amylovora in the nectary of infected flowers, we hypothesize that this feed-forward loop has evolved to enable the temporary rapid increase of flagellar motility under those specific environmental conditions. This temporary shift in motility could then be rapidly switched off again following invasion of host tissues.

We identified Lrp in a forward genetic screen that also identified additional genes as candidate negative regulators of motility in the *ΔarcZ* mutant genetic background. Among these were the diguanylate cyclase *edcB* and a gene in the cellulose biosynthetic operon. Previous work has linked *edcB* to regulation of cellulose biosynthesis in E. amylovora (11), and cellulose has been shown to physically interrupt flagellar rotation, thereby reducing motility (68). Although Hfq-dependent sRNAs have not yet been linked to cellulose biosynthesis in E. amylovora, sRNAs, including ArcZ, are known to modulate the biosynthesis of the exopolysaccharides amylovoran and levan (33, 34). It remains to be determined whether Hfq-dependent sRNAs play a role in either regulation of cyclic-di-GMP levels or in regulation of cellulose biosynthesis in E. amylovora.

Together, our data support the model proposed in Fig. 10, in which ArcZ, Lrp, and FlhDC form an incoherent feed-forward loop, and FlhDC, the final target of this feed-forward loop, regulates motility and affects levansucrase as outputs. Additionally, the intermediate, Lrp, also strongly represses amylovoran biosynthesis, and Lrp also participates in the general regulation of virulence and virulence-associated traits. Despite the fact that Lrp and FlhDC have been long characterized as regulators of fairly specific and specialized sets of genes, our work and other recent findings in Lrp (37) and FlhDC (58) suggest that these transcription factors are tied to the regulation of several virulence factors. The responsiveness of Lrp to nutrient abundance by binding directly to the amino acid leucine suggests a mechanism whereby E. amylovora may be modulating virulence-associated traits in response to nutrient availability. The reduced virulence of the Δ*lrp* mutant in the apple shoot model of infection but not in the immature pear infection model indicates that this cascade, with both Lrp and ArcZ, is required for proper regulation of E. amylovora behaviors during host infection.

## MATERIALS AND METHODS

### Culture conditions, growth, and plasmids.

Cells were routinely grown in LB medium (10 g liter^−1^ tryptone, 5 g liter^−1^ yeast extract, 5 g liter^−1^ sodium chloride). Erwinia amylovora strains were routinely grown at 28°C, and Escherichia coli strains were routinely grown at 37°C. When appropriate, antibiotics were used in the following concentrations: ampicillin, 100 μg ml^−1^; gentamicin, 10 μg ml^−1^; chloramphenicol, 10 μg ml^−1^; kanamycin, 30 μg ml^−1^. Tables of strains and plasmids used in this study can be found in Table S1 and oligonucleotides can be found in Table S2 in the supplemental material.

10.1128/mBio.00757-19.3 TABLE S1 Strains and plasmids used in this work. Download Table S1, PDF file, 0.1 MB. Copyright © 2019 Schachterle and Sundin. 2019 Schachterle and Sundin This content is distributed under the terms of the Creative Commons Attribution 4.0 International license.

10.1128/mBio.00757-19.4 TABLE S2 Oligonucleotides and primers used in this work. Download Table S2, PDF file, 0.1 MB. Copyright © 2019 Schachterle and Sundin. 2019 Schachterle and Sundin This content is distributed under the terms of the Creative Commons Attribution 4.0 International license.

### Swimming and swarming motility assays.

Swimming and swarming motility assays were conducted in soft agar medium as described previously (46). Briefly, for swarming motility, 2 μl of cells grown overnight in LB and normalized to an optical density at 600 nm (OD_600_) of 0.2 were dripped onto the surface of swarm agar plate (10 g liter^−1^ tryptone, 5 g liter^−1^ sodium chloride, 3 g liter^−1^ agar) and incubated at 28°C for 48 h and then imaged. To quantitatively assess swimming motility, cells grown overnight in LB broth were normalized to an OD_600_ of 0.2 and stab inoculated in swimming motility plates (10 g liter^−1^ tryptone, 5 g liter^−1^ sodium chloride, 2.5 g liter^−1^ agar) and incubated at 28°C for 24 h and then photographed. The area covered by swimming and swarming cells was quantified using ImageJ (69).

### Transposon mutagenesis and screen.

Transposon mutagenesis was conducted by biparental mating between the E. amylovora Ea1189Δ*arcZ* mutant strain and Escherichia coli strain S17-1 carrying Tn*5*-B20 (70) as described previously (71). Tn insertion mutants were selected on solid medium containing both ampicillin and kanamycin. For screening, mutant colonies were stab inoculated into swimming motility agar with a toothpick and incubated at 28°C for 24 h. Mutants showing a visible increase in swimming motility were aspirated from swimming motility medium and isolated to single colonies, which were then retested for swimming motility quantitatively.

### Arbitrary PCR identification of Tn insertion sites.

Identification of Tn insertion sites was conducted for Tn mutants with consistently increased motility relative to the parental Δ*arcZ* mutant strain using an arbitrary PCR approach as described previously (72), using oligonucleotides with sequences as appearing in Table S2. Following arbitrary PCR amplification of regions flanking the Tn insertion, these amplicons were sequenced by Sanger sequencing, and the resulting sequence was used with BLAST against an E. amylovora strain ATCC 49946 genome database (73) to identify the insertion site. Arbitrary PCR and sequencing were conducted from both sides of the transposon, and only strains with agreeing insertion sites were included.

### RNA isolation and qPCR.

RNA was extracted from E. amylovora cells grown overnight in liquid LB, diluted to an optical density of 0.05, and grown for an additional 6 h in LB. Cells were collected by centrifugation, and then RNA was extracted using a boiling lysis method (74). RNA quality was ensured by visualizing rRNA bands via agarose gel electrophoresis. cDNA was synthesized from 1 μg of total RNA using random hexamers and the High Capacity cDNA synthesis kit (Invitrogen, Carlsbad, CA, USA) according to the manufacturer’s instructions. Quantitative real-time PCR was conducted using 2× SYBR green master mix and run on a StepOnePlus instrument (Applied Biosystems, Foster City, CA, USA). Oligonucleotides used as primers can be found in Table S2. *recA* was used as an endogenous control, and the threshold cycle (2^−ΔΔ^*^CT^*) method (75) was used to quantify the relative abundance of transcripts.

For RNA stability testing, cultures were treated with rifampin at a final concentration of 500 μg/ml, and samples were collected immediately and at subsequent time points. Total RNA was isolated from samples, and 100 ng of total RNA was used for cDNA synthesis as described above. cDNA samples were used for qRT-PCR analysis of *lrp*, in which the *C_T_* of the sample taken immediately at the addition of rifampin was used to set 100% mRNA remaining.

### 5′ RACE assay and cloning.

We mapped the *lrp* transcriptional start site using a 5′ RACE approach (76). Briefly, total RNA was treated with *rppH* (New England BioLabs, Ipswich, MA, USA) and ligated to an RNA linker using T4 RNA ligase 1 (New England BioLabs, Ipswich, MA, USA). Linker-ligated RNA was used for cDNA synthesis with random hexamers, and the cDNA served as the template with one primer in-frame with the *lrp* coding sequence and the other matching the RNA oligonucleotide linker. PCR products were separated on a 1% (wt/vol) agarose gel, and the band with increased intensity relative to a no-enzyme control was excised, purified, and amplified. Purified products were sequenced by Sanger sequencing to determine the 5′ transcriptional start site of the *lrp* gene. A translational fusion was generated by amplifying the *lrp* 5′ UTR and the first 33 amino acids of Lrp using tagged primers and cloned in-frame with a copy of *gfp* lacking a start codon in plasmid pXG20sf (76) using an *in vivo* assembly approach as described previously (77).

The *lrp* promoter fusion was generated by cloning the 500 bases upstream of the *lrp* transcriptional start site and upstream of a promoter-less *gfp* in plasmid pPROBE-NT (78); for complementation, *lrp* with the 500 bases upstream of the start codon was cloned into plasmid pBBR1MCS-2 (79).

### Translational and transcriptional fusion assay.

Wild-type and Δ*arcZ* mutant strains carrying either the *lrp* translational fusion or the *lrp* promoter fusion were grown overnight in LB, diluted to an optical density of 0.05, and grown an additional 24 h. A sample of 100 μl of culture was then placed in the well of a 96-well plate and measured for absorbance at 600 nm and GFP fluorescence with excitement at 488 nm and emission detection at 435 nm using Tecan Spark plate reader (Tecan, Männedorf, Switzerland). GFP fluorescence was normalized to the OD_600_.

### Levansucrase assay and colony morphology assessment in the presence of 5% sucrose.

Quantification of levansucrase activity was conducted as described previously (80). Briefly, culture supernatants were mixed in a 1:1 ratio with 2 M sucrose in a phosphate-buffered solution and incubated with shaking at 28°C for 24 h, and then the resulting turbidity was measured by absorbance at 600 nm. Turbidity reported was normalized to the OD_600_ of the culture from which the supernatants were derived.

Cell morphologies were assessed from strains grown as colonies originating from single cells on solid LB medium amended with 5% sucrose. Petri plates were incubated inverted at 28°C and imaged at the various time points using an M165C dissecting scope (Leica Microsystems, Wetzlar, Germany) and a DFC295 camera (Leica Microsystems, Wetzlar, Germany).

### Amylovoran assay.

Quantification of amylovoran biosynthesis was conducted as described previously (81). Briefly, cells were grown in MBMA medium with 1% sorbitol [per liter: 3 g KH_2_PO_4_, 7g K_2_HPO_4_, 1 g (NH_4_)_2_SO_4_, 2 ml glycerol, 0.5 g citric acid, 0.03 g MgSO_4_, and 10 g sorbitol] for 48 h, after which, culture supernatants were mixed with cetylpyridinium chloride to a final concentration of 2.5 mg/ml to precipitate acidic polysaccharides. The resulting turbidity was measured as the absorbance at 600 nm. This turbidity was then normalized to the OD_600_ of the cultures grown in MBMA medium.

### Biofilm assay.

Biofilm formation was assessed using a crystal violet staining approach in a 96-well plate format as described previously (82). Briefly, following growth in the 96-well plate, planktonic cells were removed, and adherent cells were heat fixed to the microtiter plate. Adherent cells were stained with 1% crystal violet, and excess dye was washed away. Bound dye was solubilized in an 80% ethanol 20% acetone solution, and the resulting 595-nm absorbance was measured and normalized to the OD_600_ of the culture before removal of planktonic cells.

### Hypersensitive response assay.

Hypersensitive response was assessed as described previously (34) by infiltrating cells at a density of 5 × 10^7^ CFU ml^−1^ into leaves of 8-week-old Nicotiana tabacum and assessing for water soaking and cell death after 24 h.

### Immature pear and apple shoot virulence assays.

Immature pear assays were conducted as described previously (52). Briefly, immature pears were washed and sterilized using 10% bleach, after which, they were wounded and inoculated with 10^3^ to 10^4^ CFU in a 1-μl droplet and incubated at 28°C under high humidity conditions. Inoculated pears were assessed every 24 h for water soaking or necrotic symptom development.

Apple shoot virulence assays were carried out as described previously (12). Briefly, inoculum was prepared to an OD_600_ of 0.2. Sterile surgical scissors were dipped into the inoculum and used to cut between the veins of the youngest leaf of a growing shoot. The length of the necrotic lesion was measured from the point of inoculation, and measurements were made at various time points. The apple trees were 2-year-old potted trees of the cultivar Gala.
