# Supplementary figures and images for: The Leucine-Responsive Regulatory Protein Lrp Participates in Virulence Regulation Downstream of Small RNA ArcZ in Erwinia amylovora
Source: mBio. 2019 May 28;10(3):e00757-19. doi: 10.1128/mBio.00757-19 (PMC6538786; doi:10.1128/mBio.00757-19)

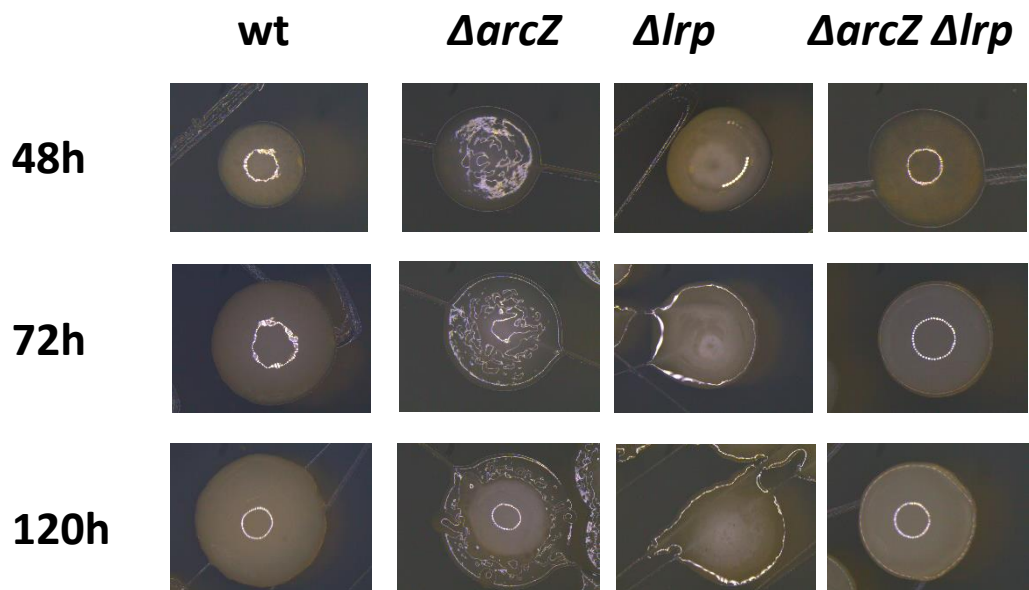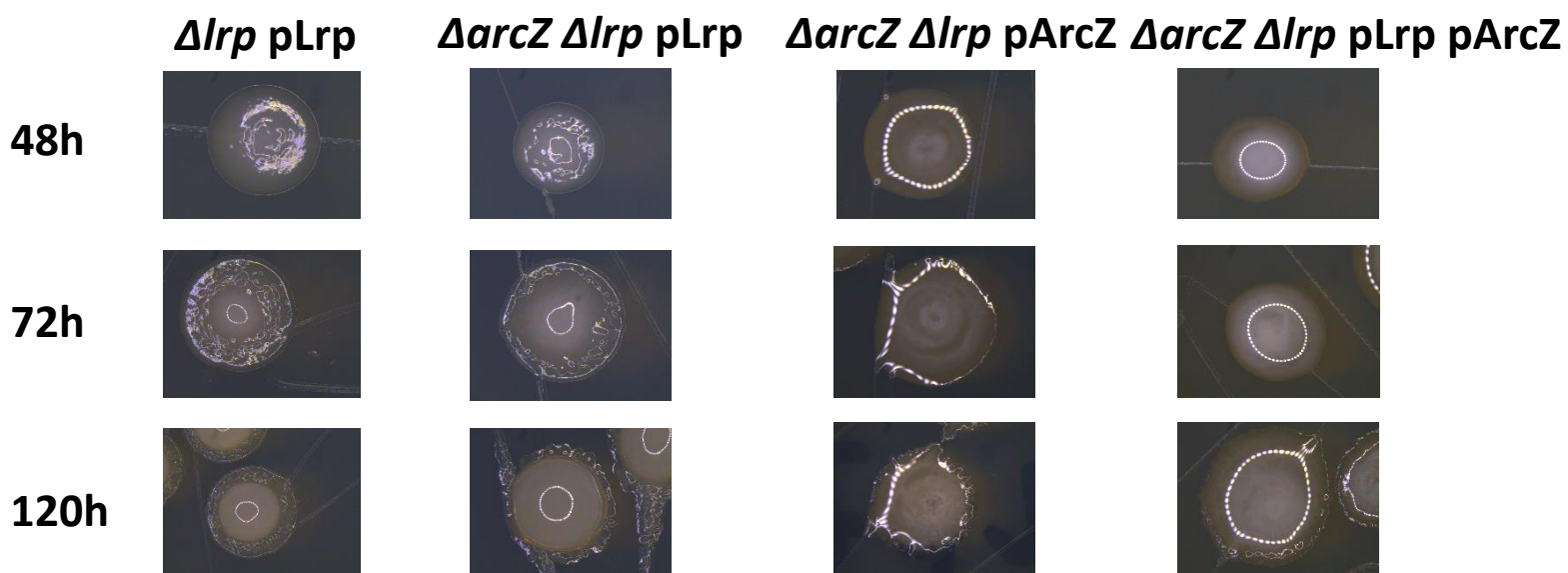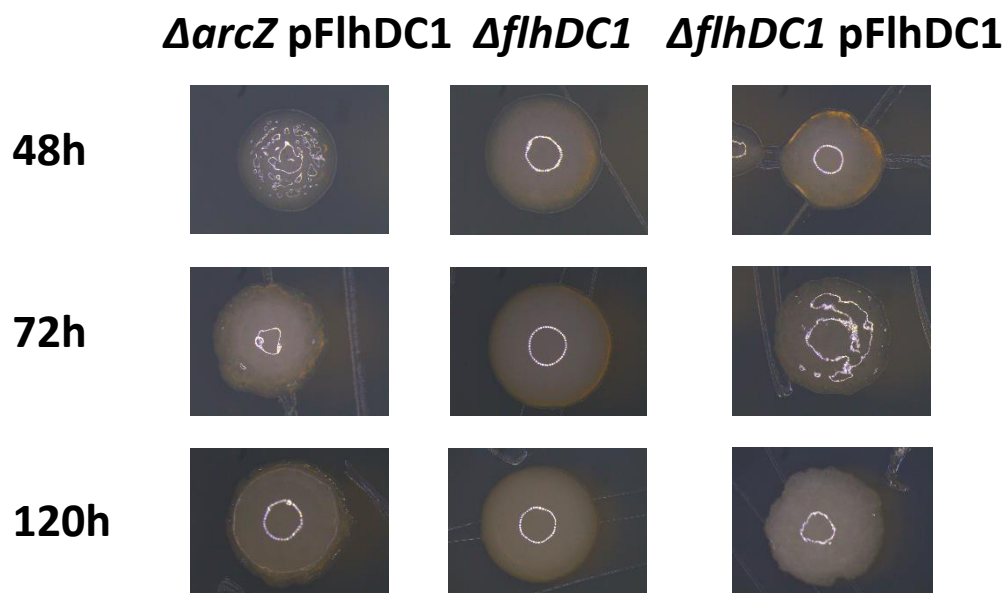

Supplement: FIG S1 [file mBio.00757-19-sf001.pdf]

**A**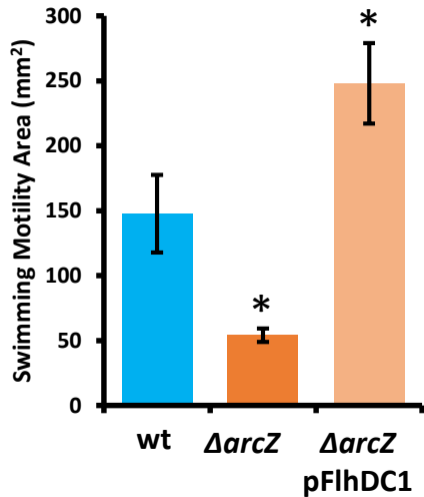**B**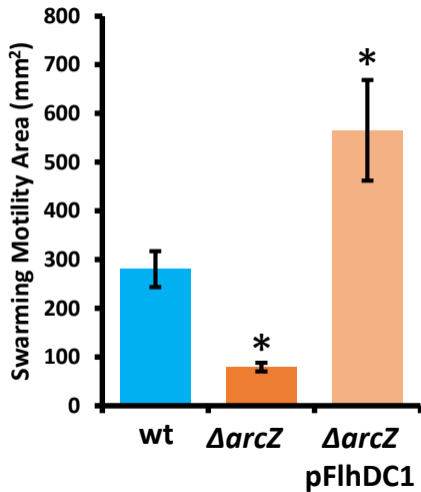

Supplement: FIG S2 [file mBio.00757-19-sf002.pdf]
