# Supplementary material for: The Leucine-Responsive Regulatory Protein Lrp Participates in Virulence Regulation Downstream of Small RNA ArcZ in Erwinia amylovora
Source: mBio. 2019 May 28;10(3):e00757-19. doi: 10.1128/mBio.00757-19 (PMC6538786; doi:10.1128/mBio.00757-19)
Supplement: TABLE S1 [file mBio.00757-19-st001.pdf]

Table S1: Strains and plasmids used in this work

Strains and Plasmids	Relevant Characteristics	Source or Reference
Escherichia coli		
DH5α		Invitrogen
S17-1	Conjugative donor for Tn mutagenesis	(Simon, Priefer, and Pühler, 1983)[1]
Erwinia amylovora		
Ea1189	wild-type	GSPB[a]
Ea1189 ΔarcZ	arcZ deletion mutant	(Zeng et al., 2013)[2]
Ea1189 Δlrp	lrpdeletion mutant	This work
Ea1189 ΔarcZ Δlrp	arcZ lrp double deletion mutant	This work
Plasmids		
pSUP102::Tn5-B20	Tn5-B20 suicide donor plasmid	(Simon, Quandt, and Klipp, 1989)[3]
pML-ArcZ	arcZ complementation	(Zeng et al., 2013)[2]
pBBR1::Lrp	lrp complementation	This work
pXG20-Lrp	lrp translational fusion	This work
pPROBE-Lrp	lrp promoter fusion	This work
pML-FlhDC1	flhDC1 complementation	This work

[a]GSPB, Göttinger Sammlung phytopathogener Bakterien, Göttingen, Germany.

[1]Simon, R. U. P. A. P., Priefer, U., & Pühler, A. (1983). A broad host range mobilization system for in vivo genetic engineering: transposon mutagenesis in gram negative bacteria. Biotechnology, 1(9), 784.

[2]Zeng, Q., McNally, R. R., & Sundin, G. W. (2013). Global small RNA chaperone Hfq and regulatory small RNAs are important virulence regulators in Erwinia amylovora. Journal of bacteriology, 195(8), 1706-1717.

[3]Simon, R., Quandt, J., & Klipp, W. (1989). New derivatives of transposon Tn5 suitable for mobilization of replicons, generation of operon fusions and induction of genes in Gram-negative bacteria. Gene, 80(1), 161-169.
