# Supplementary material for: The Leucine-Responsive Regulatory Protein Lrp Participates in Virulence Regulation Downstream of Small RNA ArcZ in Erwinia amylovora
Source: mBio. 2019 May 28;10(3):e00757-19. doi: 10.1128/mBio.00757-19 (PMC6538786; doi:10.1128/mBio.00757-19)
Supplement: TABLE S2 [file mBio.00757-19-st002.pdf]

Table S2: Oligonucleotides/primers used in this work

Identifier	Sequence	Use/Source
DGEN-1	GGCCACGCGTCGACTAGTCAGNNNNNNNNNNACGCC	Arbitrary PCR (Miller-Williams, Loewen, and Oresnik, 2006)[1]
DGEN-2	GGCCACGCGTCGACTAGTCAG	(Miller-Williams, Loewen, and Oresnik, 2006)[1]
IS50-1	CACGATGAAGAGCAGAAG	(Miller-Williams, Loewen, and Oresnik, 2006)[1]
IS50-2	TAGGAGGTCACATGGAAGTCAGAT	(Oresnik, Pacarynuk, O'Brien, Yost, and Hynes, 1998)[2]
lacZ Ext	AGGTTCCGTTCAGGACGCTACTTGTG	Arbitrary PCR
lacZ Int	CCTCCAGATCCTGGAAAACGGGAA	Arbitrary PCR
lrp knockout F	TGTAGTTCAGGGTCTGGCAGGTAAAGGGAAGAAATATATGAGAGACAC TAGTGTAGGCTGGAGCTGCTTC	Site-directed deletion of lrp
lrp knockout R	GAATTCACAGGAGTGTAGCGAAAACTATCAGGTTTTGCACCTGCCCTGT GCATATGAATATCCTCCTTA	"
lrp complementation F	CCCGACTGGAAAGCGGGCAGTGGGTGGCGTCAATCAACTGCC	In-vivo assembly cloning of Lrp with native promoter
lrp complementation R	GTTGCGTCGCGGTGCATGGGACAAGCCCGACGCTGAACC	"
pBBR1MCS F	CACTGCCCGCTTTCCAGTCGGG	In-vivo assembly amplification of pBBR1MCS-2 backbone
pBBR1MCS R	CCATGCACCGCGACGCAAC	"
Linker primer	ACTGACATGGAGGAGGGA	(Urban and Vogel, 2007)[3]
lrp RACE	GTTTTTGCTAGCGAGCTGAGCGGTATAACC	Mapping 5' transcriptional start site of lrp
lrp translational fusion F	GAGATTGACATCCCTATCAGTGATAGAGATACTGAGCACATTATAACGC GACAGGCTCTATGTC	In-vivo assembly of Lrp translational fusion
lrp translational fusion R	AGTTCTTCTCCTTTGCTCATGAATTCGCCAGAACCTTCGACGTTAGAAAT ACGTCCATC	"
pXG20 F	TGTGCTCAGTATCTCTATCACTGATAGGGATGTCAATCTC	In-vivo assembly amplification of pXG20
pXG20 R	GGTTCTGGCGAATTCATGAGCAAAGGAGAAGAACT	"
lrp qPCR F	CGCATTATCTGGACGCTTCT	
lrp qPCR R	TGTACAGCGGCGTTAAATTG	
lrp promoter F	GAGCTCGGTACCCGGGGATCCTCGGTGGCGTCAATCAACTGCC	In-vivo assembly cloning of Lrp promoter
lrp promoter R	CGACCTGAATGGAAGCCGGCCTGCCAGACCCTGAACTACATC	"
pPROBE F	GAGGATCCCCGGGTACCGAGCTC	In-vivo assembly amplification of pPROBE-NT
pPROBE R	GCCGGCTTCCATTCAGGTCG	"
flhDC F	CATGAGCTCGAACCGAATGGTTTTGCCTA	Cloning FlhDC1 into pML123
flhDC R	GTCTCTAGAAAACGTGTGGGTGAGAGGAG	"

[1]Miller-Williams, M., Loewen, P. C., & Oresnik, I. J. (2006). Isolation of salt-sensitive mutants of Sinorhizobium meliloti strain Rm1021. Microbiology, 152(7), 2049-2059.
[2]Oresnik, I. J., Pacarynuk, L. A., O'Brien, S. A., Yost, C. K., & Hynes, M. F. (1998). Plasmid-encoded catabolic genes in Rhizobium leguminosarum bv. trifolii: evidence for a plant-inducible rhamnose locus involved in competition for nodulation. Molecular plant-microbe interactions, 11(12), 1175-1185.
[3]Urban, J. H., & Vogel, J. (2007). Translational control and target recognition by Escherichia coli small RNAs in vivo. Nucleic acids research, 35(3), 1018-1037.
